# ‘Work it out’: evaluation of a chronic condition self-management program for urban Aboriginal and Torres Strait Islander people, with or at risk of cardiovascular disease

**DOI:** 10.1186/s12913-017-2631-3

**Published:** 2017-09-26

**Authors:** Kyly Mills, Michelle L. Gatton, Ray Mahoney, Alison Nelson

**Affiliations:** 1 School of Public Health and Social Work, Queensland University of Technology, Brisbane, QLD Australia; 2 First Peoples Health Unit, Griffith University, Gold Coast, QLD Australia; 3 The Institute for Urban Indigenous Health, Windsor, Brisbane, QLD Australia; 4 Aboriginal and Torres Strait Islander Studies Unit, The University of Queensland, St Lucia, QLD Australia

**Keywords:** Aboriginal and Torres Strait Islander, Cardiovascular disease, Chronic condition, Urban Indigenous, Program evaluation

## Abstract

**Background:**

Chronic diseases disproportionately burden Aboriginal and Torres Strait Islander people in Australia, with cardiovascular (CV) diseases being the greatest contributor. To improve quality of life and life expectancy for people living with CV disease, secondary prevention strategies such as rehabilitation and self-management programs are critical. However, there is no published evidence examining the effect of chronic condition self-management (CCSM) group programs for Aboriginal and Torres Strait Islander people who have, or are at risk of, CV disease specifically. This study evaluates the *Work It Out* program for its effect on clinical outcome measures in urban Aboriginal and Torres Strait Islander participants with or at risk of CV disease.

**Methods:**

This study was underpinned by a conceptual framework based on Aboriginal and Torres Strait Islander community control. Participants had at least one diagnosed CV disease, or at least one CV disease risk factor. Short-term changes in clinical outcome measures over (approximately) 12 weeks were evaluated with a quasi-experimental, pre-post test design, using paired t-tests. Factors contributing to positive changes were tested using general linear models. The outcome measures included blood pressure (mmHg), weight (kg), body mass index (kg/m^2^), waist and hip circumference (cm), waist to hip ratio (waist cm/hip cm) and six minute walk test (6MWT).

**Results:**

Changes in several clinical outcome measures were detected, either within the entire group (*n* = 85) or within specific participant sub-groups. Participant’s 6MWT distance improved by an average 0.053 km (95% CI: 0.01–0.07 km). The change in distance travelled was influenced by number of social and emotional wellbeing conditions participants presented with. The weight of participants classified with extreme obesity decreased on average by 1.6 kg (95% CI: 0.1–3.0 kg). Participants with high baseline systolic blood pressure demonstrated a mean decrease of 11 mmHg (95% CI: 3.2–18.8 mmHg). Change in blood pressure was influenced by the number of cardiovascular conditions participants experienced.

**Conclusions:**

Short-term improvements seen in some measures could indicate a trend for improvement in other indicators over the longer term. These results suggest the *Work It Out* program could be a useful model for cardiovascular rehabilitation and prevention for other urban Aboriginal and Torres Strait Islander populations.

## Background

Chronic diseases disproportionately burden Aboriginal and Torres Strait Islander people in Australia, contributing to 80% of the mortality gap between Aboriginal and Torres Strait Islander and non-Indigenous people [1–4]. The greatest contributors to this mortality gap are cardiovascular (CV) diseases, with Aboriginal and Torres Strait Islander people three times more likely to suffer a major coronary event than other Australians [3, 5]. Since Aboriginal and Torres Strait Islander health does not just impact the physical wellbeing of an individual but also the social, emotional and cultural well-being of the whole community [6], the implications of CV diseases may extend from individuals through to their families and communities. Effective prevention and management of these conditions is elemental to reach the goal of closing the life expectancy gap between the Aboriginal and Torres Strait Islander population and non-Indigenous Australians within a generation [7].

In order to improve quality of life and life expectancy for Aboriginal and Torres Strait Islander people living with CV disease, secondary prevention strategies such as cardiac rehabilitation and self-management are critical. Published evidence for best practice CV rehabilitation and self-management indicate key features of effective programs. These include approaches that understand health and wellness through an Aboriginal and Torres Strait Islander lens and ensure an holistic approach with care delivered by a multidisciplinary team that includes Aboriginal and Torres Strait Islander Health Workers (AHWs) or other Aboriginal and Torres Strait Islander care providers [8–12]. Further, secondary prevention for CV disease should include physical activity and education that is delivered, where possible, in a group or family setting [13–16] and within a culturally responsive environment, such as an Aboriginal and Torres Strait Islander Community Controlled Health Service (ATSICCHS) setting [11, 12] and implement strategies that address a range of chronic conditions and comorbidities where appropriate [10–12, 17]. Evaluated CV disease specific, culturally appropriate group programs for Aboriginal and Torres Strait Islander people that incorporate at least some of these evidence-based strategies, show some short term improvements in CV disease risk factor measures [11, 12]. However, documented CV specific group programs for Aboriginal and Torres Strait Islander peoples are rare.

The implementation and evaluation of broad chronic condition self-management (CCSM) programs are more common in the literature, and have been delivered in Aboriginal and Torres Strait Islander settings. Although not CV specific programs, many people who participate present with CV disease and other comorbidities and/or multiple risk factors for CV disease. Correspondingly, such programs tend to share many strategies recognised as important for CV prevention and management. The *Stanford Chronic Disease Self-Management Program* [18, 19] is a common CCSM strategy made available through the Australian healthcare system in 2004. In a study evaluating the model in an Aboriginal and Torres Strait Islander community, improvements were noted in some CV risk factor measures including BMI, cholesterol levels and glycosylated haemoglobin [20]. The *Flinders Program* is also a widely adopted CCSM program in clinical settings throughout Australia [21–24]. When implemented and evaluated at three community controlled health settings in rural, regional and metropolitan South Australia, the *Flinders Program* along with other coordinated systemic CCSM strategies showed small but statistically significant improvements over time in CV risk factor indicators of BMI, total cholesterol, triglyceride, low-density lipoprotein, and glycosylated haemoglobin levels [24] .

There is undoubtedly a place for cardiac rehabilitation and self-management to occur within broader group programs for CCSM, as these adopt similar CV-specific strategies, address similar risk factors, and have indicated some improvements across CV risk factor indices. However, there is no published evidence examining the effect of such broader CCSM group programs for urban Aboriginal and Torres Strait Islander people who have, or are at risk of, CV disease specifically. The *Work It Out* program is a novel approach to CCSM underpinned by the aforementioned evidenced-based strategies that has been developed and implemented by the *Institute for Urban Indigenous Health (IUIH).* This paper aims to evaluate the program for its effect on clinical outcome measures in a population of Aboriginal and Torres Strait Islander participants with or at risk of CV disease in urban south-east Queensland.

### Program overview

The *Work It Out* Program is a culturally responsive CCSM group program that has been implemented since 2011 across eight ATSICCHSs in south-east Queensland. Adopting the holistic view of Aboriginal and Torres Strait Islander health, the *Work It Out* program combines an inter-professional allied-health partnership approach based on published evidence of best practice CCSM and rehabilitation strategies with Aboriginal and Torres Strait Islander people.

Entry to the program is by General Practitioner referral at participating ATSICCHSs, with many participants referred for the prevention or management of CV conditions. The progam runs on a successive 12 week cycle, with clients able to participate in two or more *Work It Out* sessions per week (as dictated by participant and location demands). The program has flexible entry and exit points and allows participants to be absent from several sessions due to responsibilities within family and community, and then return to the program at a later time. A *Work It Out* session consists of a 45 min ‘yarning’ (education) session delivered by a variety of health professionals in a culturally-safe environment. This is followed by a one hour exercise program undertaken in a group setting, developed by an accredited exercise physiologist or physiotherapist and indivudally tailored to meet the needs of participant’s unique chronic conditions. An AHW or other Aboriginal and Torres Strait Islander staff member is an integral part of the team working closely with the accredited exercise physiologist/physiotherapist at each site.

## Methods

This study was underpinned by a conceptual framework based on the principle of Aboriginal and Torres Strait Islander community control. This conceptual framework began with the research design, priorities and direction being set and monitored by a community controlled health organisation, through a relationship of trust developed over time. Two authors (KM, RM) are Aboriginal researchers, ensuring the research was culturally and ethically responsive, and driven by Aboriginal and Torres Strait Islander priorities. The lead author was employed with the *Work It Out* program and developed relationships with participants and colleagues over a 12 month period, before this study took place. Additionally, an upper-management member of the IUIH staff was part of the research team for this project (AN). This ensured ongoing support of the project that aligned with the IUIH core values. Persistent guidance throughout the entire research process, including data collection, analysis and dissemination was provided by *Work It Out* staff. Significantly, *Work It Out* participants became part of the research process, through the attendance at yarning sessions by the researcher to informally share research outcomes. Frequently, this would involve a reciprocal learning process whereby participants would ask questions of the research that would require further investigation, and regular reporting back to their *Work It Out* group. It is this framework that shaped the direction of the research project which became imperative for conducting ethical and culturally competent quantitative research with the population group. This conceptual framework aligned to the *National Health and Medical Research Council’s* guidelines for ethical conduct in Aboriginal and Torres Strait Islander research [25] and shaped ethical research practice, ensuring that Aboriginal and Torres Strait Islander people and perspectives were included in the collection, analysis and dissemination of the research [25–29].

A quasi-experimental, pre-post test design was used to test short-term changes in routinely collected clinical outcome measures, following participation in the *Work It Out* program, from baseline to first follow-up assessment, for all participants with or at risk of CV disease. The research hypothesis was that participation in the WIO program is associated with short-term stable or improved clinical outcome measures for participants with or at risk of CV disease.

A secondary outcome of this research was to explore factors that may have influenced positive changes in these clinical outcome measures, to provide a quantitative understanding about elements which may contribute to the *Work It Out* participant’s overall wellbeing. This method was specifically chosen as there is a pressing identified need to ensure research questions are developed in a culturally competent manner, considering methods that move away from a narrative of deficit and disadvantage, and instead, seek to empower wellness within Aboriginal and Torres Strait Islander communities [26, 30, 31].

### Participants

Participants were recruited purposively from a population of urban Aboriginal and Torres Strait Islander patients (18 years or over) with or at risk of chronic disease, who attended one of six participating ATSICCHSs in South-east Queensland between 2012 and 2014. Participants were referred by their general practitioner into the program, after completion of an *Aboriginal and Torres Strait Islander Health Assessment* [32]*, General Practitioner Management Plan* and/or *Team Care Arrangement* [33]*.*

Inclusion criteria for this study specified that participants have at least one diagnosed CV disease, or at least one CV disease risk factor. Participants were included in the study if they had any existing CV disease affecting the heart and/or blood vessels [34, 35]. Participants existing CV diseases were diverse and included: congestive heart failure/chronic heart failure; cardiomyopathy; angina; myocardial infarct/acute myocardial infarct; arrhythmia; valvular disease; ischemic heart disease; cerebrovascular disease/stroke; deep vein thrombosis; pulmonary embolism; and, peripheral vascular disease*.* Additionally, participants were included in the study if they had at least one of the following risk factors for CV disease: the presence of diabetes mellitus or insulin dependent diabetes mellitus; high cholesterol; hypertension; overweight/obesity; the presence of a social and emotional wellbeing condition (SEWB); chronic kidney disease; having a heavy alcohol intake; being a current smoker [5, 36, 37]; and, being over 74 years of age at baseline [38].

### Data

Upon entry to the *Work It Out* program, an initial assessment is routinely undertaken by an accredited exercise physiologist or physiotherapist where basic baseline demographic and medical history are collected, as well as baseline outcome measures. At this initial assessment, participants were counselled in plain English about the research project and only those who gave signed, informed consent were included.

The collection of medical history at baseline determined participants existing CV conditions and risk factors for CV disease which allowed for the decision of inclusion into to the study using the aforementioned inclusion criteria. This medical history was cross-referenced with data from the participants most recent *Aboriginal and Torres Strait Islander Health Assessment*, within patient medical files. Descriptive data, such as the number and presence of other co-morbidities were also collected from these sources. Baseline clinical outcome measures included systolic and diastolic blood pressure (BP) (mmHg); weight (kg) and body mass index (BMI) (kg/m^2^); waist, hip circumference (cm) and Waist Hip Ratio (WHR) (waist cm/hip cm); and, six minute walk test (6MWT). Systolic and diastolic BP was measured using an electronic sphygmomanometer on participants in the seated position. Weight (kg) was measured without shoes, using an electronic body mass scale. Height (cm) was measured using a tape measure from floor to crown of head, and corresponding BMI calculated (BMI = kg/m2). Waist circumference (cm) was taken using a tape measure at midpoint between the iliac crest and lower ribs. Likewise, hip circumference (cm) was measured with a tape measure at the maximum point of prominence of the buttocks. WHR was calculated using weight and hip circumference measurements (WHR = Waist circumference/hips circumference). The 6MWT measures the distance an individual can comfortably walk in 6 min and was undertaken following the standard *American Thoracic Society Guidelines* [39].

These clinical outcome measures were also collected at six week intervals throughout the program. Whilst the aim was to collect follow-up clinical outcome data after a 12 week period, the rolling program entry and flexibility in attendance meant the timing of follow up physical assessments varied between participants. Missing follow-up data occurred if participants were absent from the program on the day the assessments were scheduled, or, if they had discontinued the program. Furthermore, due to resourcing and/or time limitations, often not all clinical outcome measures were recorded for each participant at each follow-up session. Only participants who recorded at least one clinical outcome measure at their initial and first follow-up (approx. 12 weeks) assessment were included for analysis. All data were de-identified onsite at *IUIH* and provided for the purposes of secondary data analysis.

### Statistical analysis

Paired t-tests were used to determine if there was a statistically significant change in clinical outcome measures between baseline and follow-up assessment. In addition to the total cohort, participant sub-groups were allocated during secondary data analysis using the following published evidence-based criteria which was determined a priori:

- BMI groups – *healthy*, *overweight*, *obese I*, *obese II* and *extreme obesity* [40];
- BP categories – *normal*, *high/normal*, *high*, *very high* [41];
- Waist and WHR groups – *no increased risk of metabolic complications, increased risk* and *substantially increased risk* [1, 40].

Clinical groups were assigned based on the participant’s baseline measurement.

Change in each clinical outcome measure, and potential factors influencing the change were explored using general linear models. Models were built by examining the significance of factors and covariates individually, or in combination. Factors considered were BMI group, presence of CV disease, number of CV conditions, presence of respiratory disease, presence of diabetes mellitus/insulin dependent diabetes mellitus, number of SEWB conditions, presence of a musculoskeletal condition, number of chronic conditions, number of CV risk factors and gender. Presence of DM/IDDM, musculoskeletal, respiratory disease and number of SEWB conditions were chosen because of their overrepresentation within this population. SEWB is often used interchangeably with ‘mental health’, however, is a much more multifaceted concept with particular meaning for Aboriginal and Torres Strait Islander people [42]. While the term SEWB includes mental health conditions, it also encompasses broader aspects of health and wellbeing such as connection to land or Country, culture, family, kinship and community [42]. Covariates considered included number of days between baseline and follow-up measurement, number of visits between baseline and follow-up, average sessions attended per week, baseline measurement and age. A type I error of less than 0.05 was considered to represent statistical significance for all tests. Due to the small sample size, a maximum of 2 independent variables were included in each model, with those selected having the highest explanatory power. Co-linearity between variables was assessed using the Variance Inflation Factor (VIF); all results had a VIF < 1.4 indicating co-linearity is not a problem in the models. All analysis were performed using *IBM SPSS for Windows Release 22 (SPSS)*.

As this is a small and potentially recognisable cohort of participants, confidentiality of study participants must be upheld. Thus, the actual sample size of two sub-groups in each clinical outcome measure have been replaced with the labels “*n* ≤ 10” or “*n* ≥ 10” when reporting outcomes. Statistical tests were not conducted on sub-groups where *n* ≤ 10.

## Results

A total of 315 participants had baseline clinical outcome measurements recorded upon initial assessment by an accredited exercise physiologist or physiotherapist. Of this sample, 85 people with (*n* = 24) or at risk (*n* = 61) of CV disease had at least one paired clinical outcome baseline and follow-up measurement recorded, thus excluding 230 participants from analysis.

Participant characteristics for the sample who had only a baseline measurement recorded (*n* = 230) were compared to those for participants with both baseline and follow-up measurement (*n* = 85). Those that had a paired baseline and follow-up measurement were an average of 5.14 years older (95%CI: 1.56 to 8.73) than those that had a baseline measurement only (*t*312 = 2.82, *p* = 0.005). Participants did not differ between groups in gender, marital status, employment status or baseline CV disease risk factor characteristics.

For the sample included in this study (n = 85), presenting with more than one CV condition was common, with 11 (45.85%) participants having 2–3 CV conditions. The median length of time between baseline and follow-up assessment was 11.85 weeks (IQR = 6.57–26.64 weeks) and the median number of sessions attended per person during this period 11.00 (IQR = 6.00–19.00). The mean age of participants was 55.26 years (SD = 13.86 years) and the majority (*n* = 61, 71.80%) were female. SEWB conditions were common amongst the 85 participants, with 35 (41.20%) presenting with one SEWB condition and 13 (15.30%) having two-three SEWB conditions.

At baseline 76.54% of the participants with both a pre and post measurement were classified as obese or extremely obese. The mean BMI was 36.89 (SD = 9.65). Almost one third of participants (*n* = 18) had high systolic BP and 91.14% had a waist circumference categorised as having substantially increased risk of metabolic complications. There was high variability in the distance walked in the 6MWT, with participants covering an average of 0.38 km and individual values ranging from 0.07 to 0.95 km.

At follow-up participants with matched baseline line data showed small, non-significant decreases in mean weight, BMI, waist circumference, hip circumference, systolic BP, and diastolic BP, and a slight increase in WHR between baseline and follow-up (Table 1). The distance walked in the 6MWT significantly increased between baseline and follow-up with a mean change of 0.053 km (95% CI: 0.01 to 0.07, *p* = 0.023) (Table 1). Significant changes were identified for specific participant sub-groups. The *extreme obesity* BMI group demonstrated a significant reduction in weight of 1.57 kg (95% CI: 0.10 to 3.03, *p* = 0.037)*.* For both systolic and diastolic BP, participants with a baseline measurement recorded in the *normal* range, had a significantly higher measurement at follow-up. For systolic BP only, participants in the *high* range at baseline demonstrated a significant decrease at follow-up of 11 mmHg (95% CI: 3.18 to 18.82, *p* = 0.009) (Table 1).

**Table 1:** Summary of changes in clinical outcome measures for individuals with baseline and first follow-up assessment

Clinical Outcome Measure	*n*	Mean Change (95% CI)	Baseline Measurement (SD)	Follow-up Measurement (SD)	*p-value†*
Weight (kg)	65	−0.57 (−1.28, 0.14)	98.90 (27.97)	98.32 (27.65)	0.113
Weight within BMI Groups (kg)					
*Healthy*	≤10	0.26 (−3.30, 3.82)	66.53 (7.84)	66.80 (8.13)	–
*Overweight*	≥10	−0.02 (−1.12, 1.08)	76.35 (9.76)	76.33 (10.40)	0.974
*Obese I*	15	−1.21 (−3.11, 0.69)	92.39 (12.37)	91.17 (11.11)	0.194
*Obese II*	12	0.85 (−0.46, 2.15)	95.64 (9.67)	96.48 (9.67)	0.181
*Extreme Obesity*	19	−1.57 (−3.03, −0.10)	130.55 (27.23)	128.98 (28.09)	0.037*
BMI (kg/m^2^)	64	−0.20 (−0.47, 0.07)	35.85 (8.92)	35.65 (8.80)	0.15
Systolic BP (mmHg)	58	−0.71 (−4.85, 3.43)	128.21 (16.84)	127.50 (16.31)	0.734
Systolic BP within BP Categories (mmHg)					
*Normal*	≥10	8.19 (3.45, 12.93)	110.29 (6.41)	118.48 (10.06)	0.002*
*High/Normal*	≥10	−0.79 (−8.17, 6.59)	129.21 (4.88)	128.42 (14.82)	0.825
*High*	18	−11.00 (−18.82, −3.18)	148.06 (7.87)	137.06 (18.49)	0.009*
Diastolic BP (mmHg)	58	−0.12, (−3.49, 3.25)	80.50 (10.17)	80.38 (11.29)	0.943
Diastolic BP within BP Categories (mmHg)					
*Normal*	≥10	5.31 (1.59, 9.04)	73.63 (6.65)	78.94 (9.55)	0.007*
*High/Normal*	≥10	−5.87 (−13.48, 1.74)	85.13 (2.87)	79.25 (13.88)	0.121
*High*	9	−8.11 (−16.08, −0.14)	93.33 (4.06)	85.22 (10.12)	–
Waist circumference (cm)	60	−0.58 (−3.06, 1.89)	116.32 (19.86)	115.73 (18.98)	0.63
Waist circumference within Waist circumference groups (cm)					
*No increased risk*	≤10	5.33 (−5.01, 15.68)	93.66 (10.06)	88.33 (8.08)	–
*Increased risk*	≤10	1.77 (−14.15, 17.69)	84.07 (3.00)	85.83 (4.54)	–
*Substantially increased risk*	54	−1.05 (−3.74, 1.65)	119.7 (17.94)	118.62 (17.61)	0.44
Hip circumference (cm)	57	−0.65 (−2.55, 1.25)	123.57 (18.39)	122.9 (19.80)	0.49
WHR (waist/hip)	57	0.0007 (−0.024, 0.025)	0.94 (0.093)	0.95 (0.091)	0.95
WHR within WHR Groups (waist/hip)					
*No increased risk*	≤10	0.108 (−0.001, 0.219)	0.81 (0.022)	0.92 (0.12)	–
*Substantially increased risk*	≥10	−0.016 (−0.038, 0.004)	0.97 (0.080)	0.95 (0.086)	0.12
6MWT (km)	47	0.053 (0.01, 0.07)	0.38 (0.18)	0.43 (0.16)	0.023*

*† Paired t-test used to compare baseline and follow-up measurement. Statistical testing only conducted when n > 10 in each group*

*Significant at p < 0.05

**Fig. 1:**
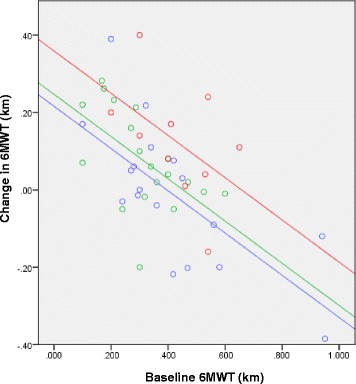
Relationship between baseline 6MWT and change in 6MWT (follow-up – baseline), and predicted regression equations. Fig. 1 legend: blue circle 0 SEWB Conditions, green circle 1 SEWB Condition, red circle 2–3 SEWB Conditions

After adjusting for baseline measurements, statistically significant models predicting positive changes in 6MWT and systolic and diastolic BP were developed. Participants who walked further in their 6MWT at baseline, saw smaller changes in their follow-up 6MWT distance. The best fitting model for change in 6MWT adjusted for baseline 6MWT, included number of SEWB conditions as a factor (Fig. 1). This model predicted that after adjusting for baseline 6MWT measurement, those individuals who had two or three SEWB conditions walked an average of an additional 0.143 km (95%CI: 0.05 to 0.23, *p* = 0.002) at follow-up, compared to those with no SEWB conditions (Fig. 1).

It was noted that participants with two or three SEWB conditions had a lower mean weight and walked substantially further at baseline (mean weight = 87.74 kg, mean baseline 6MWT distance = 0.55 km), compared to those that had one SEWB condition (mean weight = 104.33 kg, mean baseline 6MWT distance = 0.38 km) and those that presented with no SEWB conditions (mean weight = 100.83 kg, mean baseline 6MWT distance = 0.39 km).

**Fig. 2:**
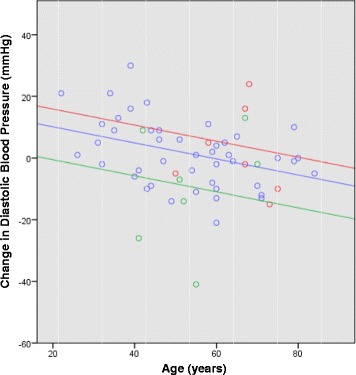
Relationship between age and change in diastolic blood pressure (follow-up – baseline), and predicted regression equations. Fig. 2 legend: blue circle 0 CV Conditions, green circle 1 CV Condition, red circle 2+ CV Conditions

Like the 6MWT, the largest decreases in systolic and diastolic BP between baseline and follow-up occurred in those participants with the highest baseline readings. After adjusting for baseline measurement, change in diastolic BP was found to be influenced by the number of CV conditions and age (Fig. 2). Participants with one CV condition experienced an average decrease in diastolic BP which was 10.6 mmHg larger (95% CI: 3.3 to 18.0 mmHg, *p* = 0.004) than participants with no CV conditions, after adjusting for baseline diastolic BP and age (Fig. 2). Similarly, the model for change in systolic BP showed that after adjusting for the baseline reading, participants with one CV condition experienced a 10.77 mmHg (95% CI:0.40 to 21.13, *p* = 0.042) larger decrease in systolic BP, when compared to those who didn’t present with a CV condition. Further, the model for diastolic BP showed that for every one year increase in age, the change in diastolic BP is expected to decrease by 0.26 mmHg (95% CI: 0.09 to 0.43 mmHg, *p* = 0.002) after adjusting for number of CV conditions and baseline diastolic BP (Fig. 2).

## Discussion

The participants in the Work it Out program are purposely selected to have at least one chronic condition or risk factor, thereby automatically selecting a portion of the larger Aboriginal and Torres Strait Islander population with poorer health. However the baseline clinical outcome measures indicate a population that has a substantial burden of ill health with multiple conditions and/or risk factors and a high level of complex health needs. It appears this urban south-east Queensland Aboriginal and Torres Strait Islander cohort has similar characteristics to cohorts accessing CV rehabilitation, prevention and CCSM programs in rural, regional and urban areas of other Australian states, specifically Tasmania, Western Australia and South Australia [11, 12, 34, 35].

The analysis undertaken during this study demonstrated that statistically significant changes in some clinical outcome measures were detected after approximately 12 weeks in the *Work It Out* program for people with or at risk of CV disease. Results indicated that changes in clinical outcome measures were seen in measures that were more responsive to exercise within a shorter time period, and in participants with a worse starting condition. This is emulated in a Tasmanian cardiopulmonary rehabilitation and secondary prevention program [12] which recognised the difficulty in achieving losses in weight, BMI and waist circumference over an eight week period, noting small effect sizes. Correspondingly, the 6MWT is proven to be more responsive to change over a shorter time period, with more modest changes seen over longer periods [43].

The reduction in systolic BP for participants in the *high* category of 11 mmHg is notable and clinically relevant, and is greater than that experienced in both a similar cohort [21] and a meta-analysis of conventional cardiac rehabilitation programs [44]. It is echoed however, in results from a similar cardiac rehabilitation program [11] which indicated that along with lifestyle changes and medication compliance, a higher measure of BP at baseline may correspond to a greater loss in the short term, as demonstrated in this study. A reduction in systolic BP may have particular clinical importance for this group, as clinical trials have shown that control of systolic hypertension specifically, reduces total mortality, CV mortality, stroke and the occurrence of other heart failure events [45, 46].

The statistical models that were created for both systolic and diastolic BP showed that both appeared to be influenced by the number of CV conditions, after adjusting for their baseline measurement. It is possible that this points towards prescribed intensity of exercise, with participants who presented with two or more CV conditions more likely prescribed a reduced exercise intensity than those who presented with one CV condition, thus potentially explaining the smaller changes at follow-up assessment. The effect of medication could also be important, with participants who presented with multiple CV conditions more likely to have their BP regulated by medication and thus show a smaller effect from exercise and lifestyle modification. Lastly, this outcome could be influenced by motivation. That is, participants with one CV condition may be more motivated to make lifestyle changes, compared to those who have numerous CV conditions, who may find it more challenging. The exact reason for the influence of number of CV conditions on change in systolic and diastolic BP in this population is an area for further research.

The statistical model also demonstrates the impact of age on diastolic BP. It is likely this is due to the natural progression of diastolic BP, which rises until approximately age 50 and tends to level off or fall in later life [47, 48].

The 15% improvement in 6MWT is consistent with what is noted in conventional short-term outpatient cardiac rehabilitation programs [49] and points to the effect of regular exercise on every day physical functioning. The model indicated that change in 6MWT appeared to be influenced by number of SEWB conditions, with the overall pattern of participants who walked further at baseline seeing the smallest improvement at follow-up. However, this trend did not hold for those experiencing several SEWB conditions who also had a smaller mean weight compared to those with one or no SEWB conditions. This may suggest that those participants with two or three SEWB had the ability to walk further at baseline because they were leaner and thus more physically able. However, while weight is likely to influence the 6MWT result at baseline, it cannot explain the differential improvements in 6MWT at follow-up. What this may reveal then, is the social benefit gained from attending the program and/or the physiological improvements in depressive symptoms that are often associated with exercise.

The presence of psychosocial stress is linked to a lack of motivation in carrying out work or normal activities for Aboriginal and Torres Strait Islander people [50]. Hence, we propose that participants who began the program with two or three SEWB conditions, could have been significantly lacking in motivation, and that their superior performance in the 6MWT at baseline compared to other participants was due to their better physical condition (i.e. lighter body weight). A separate qualitative study involving a small sample of *Work It Out* participants (*n* = 22), identified social connectedness as a strong theme, with reports of “having a place to meet others,” connecting and re-connecting with family and friends and a shared responsibility to help each other emphasised [51]. So, we hypothesise that as participants with numerous SEWB conditions continued to attend the program, they benefited from this important social support, thus increasing motivation levels. This increase in motivation may have manifested as a greater change in the 6MWT, as this is proven to be highly responsive to exercise in a short term period [43]. Moreover, qualitative analysis revealed overwhelming reports of decreases in anxiety and depression in participants due to participation in the program [51]. This is supported by literature which shows that for participants with diagnosed depression, physical exercise in a group setting may substantially increase 6MWT distance, improve quality of life and depressive symptoms, and increase perceptions of social support when compared to an intervention of traditional cognitive behavioural therapy [52, 53]. This highlights the potential physiological effects of exercise for participants with numerous SEWB conditions.

Although the distance walked in six minutes may be seen as an objective measure of physical capacity, the potential flow-on effects for individuals with two or three SEWB could be considerable. It is important too, to examine the meaning of this physical increase in 6MWT in the context of Aboriginal and Torres Strait Islander people participating in a community based health program. For Aboriginal and Torres Strait Islander people, walking an extra 143 m may be reflected in an increase in the ability to physically participate more wholly in community, interact with family and carry out cultural activities. More research determining the exact effects of the *Work It Out* program on participants with SEWB conditions is warranted.

This study has several limitations which need to be considered. First, only changes that occurred over an approximate 12 week period were assessed. Further analysis is required over a longer time period in order to see if current benefits are sustained and/or positive trends in changes to clinical outcome measures continue. Second, the bivariate results of this study are likely impacted by regression to the mean and should be viewed with caution. However, the impact of regression to the mean was reduced in the modelling but the inclusion of the baseline measurement in the model. Hence the modelling results reported are more likely representative of true covariate effects compared to the simpler bivariate analysis. Further, the statistical analysis and power was restricted due to the small sample size, particularly in certain sub-groups. This is a result of the *Work It Out* program being relatively new and also a lack of follow-up data. It is recommended that a similar analysis be conducted once more participants have progressed through the program. Furthermore, the exclusion of participants without a follow-up assessment may offer some bias to results. However, as age is the only characteristic identified that differs between the included and excluded cohorts, this is unlikely to be significant. As an observational study, this project is subject to the potential limitations of this study design. These include a lack of control group and an inability to establish definitive cause and effect relationships. However, the overall outcome is the impact of the *Work It Out* program and how this has influenced behaviour change in participants’ everyday lives, so an observed positive change is deemed reflective of the program’s success. Lastly, the diversity of Aboriginal and Torres Strait Islander communities must be considered, and thus, caution must be exercised when generalising these outcomes beyond urban south-east Queensland.

## Conclusions

Evaluation of the *Work It Out* program over an approximate 12 week period found statistically significant improvements for those participants who began the program with high systolic BP; those whose BMI was classified as extremely obese; and, in the 6MWT. These findings suggest that short-term improvements were seen in those clinical outcome measures that were more responsive to exercise and behaviour change within shorter time periods. This could indicate a trend for improvements in other clinical outcome indicators over the longer term. It was noted that there were increased benefits with particular to those participants suffering numerous SEWB conditions and the reasons for this require further investigation. Whilst evaluation of other aspects of the *Work It Out* program are required, this study shows that the program could prove a useful model for CV rehabilitation and prevention for other urban Aboriginal and Torres Strait Islander populations.

## Abbreviations

6MWT: Six Minute Walk Test
AHW: Aboriginal and Torres Strait Islander Health Worker
ATSICCHS: Aboriginal and Torres Strait Islander Community Controlled Health Service
BMI: Body Mass Index
BP: Blood Pressure
CCSM: Chronic Condition Self-Management
CV: Cardiovascular
IQR: Interquartile Range
IUIH: The Institute for Urban Indigenous Health
SEWB: Social and Emotional Wellbeing
WHR: Waist to Hip Ratio
